# Supplementary material for: BUB1 Promotes Gemcitabine Resistance in Pancreatic Cancer Cells by Inhibiting Ferroptosis
Source: Cancers (Basel). 2024 Apr 18;16(8):1540. doi: 10.3390/cancers16081540 (PMC11048608; doi:10.3390/cancers16081540)

Figure 2F&2H

Pan1（n E50）MIA(N E50)

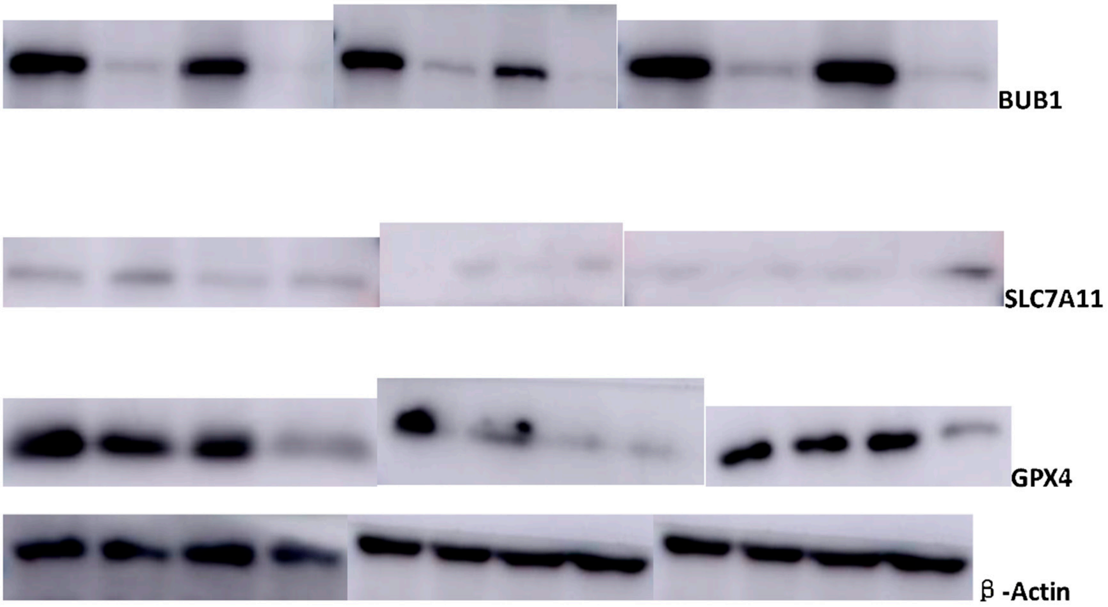

Figure 3

Figure 3B

Bub1(pan1)

Actin(pan1)

Bub1(mia)

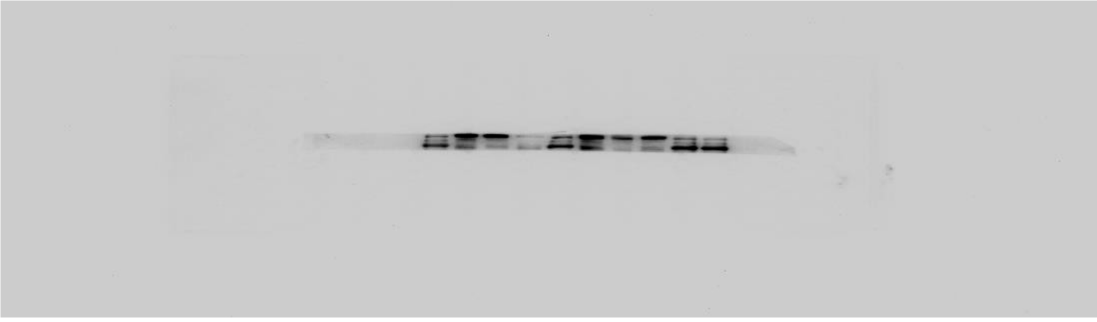

Actin(mia)

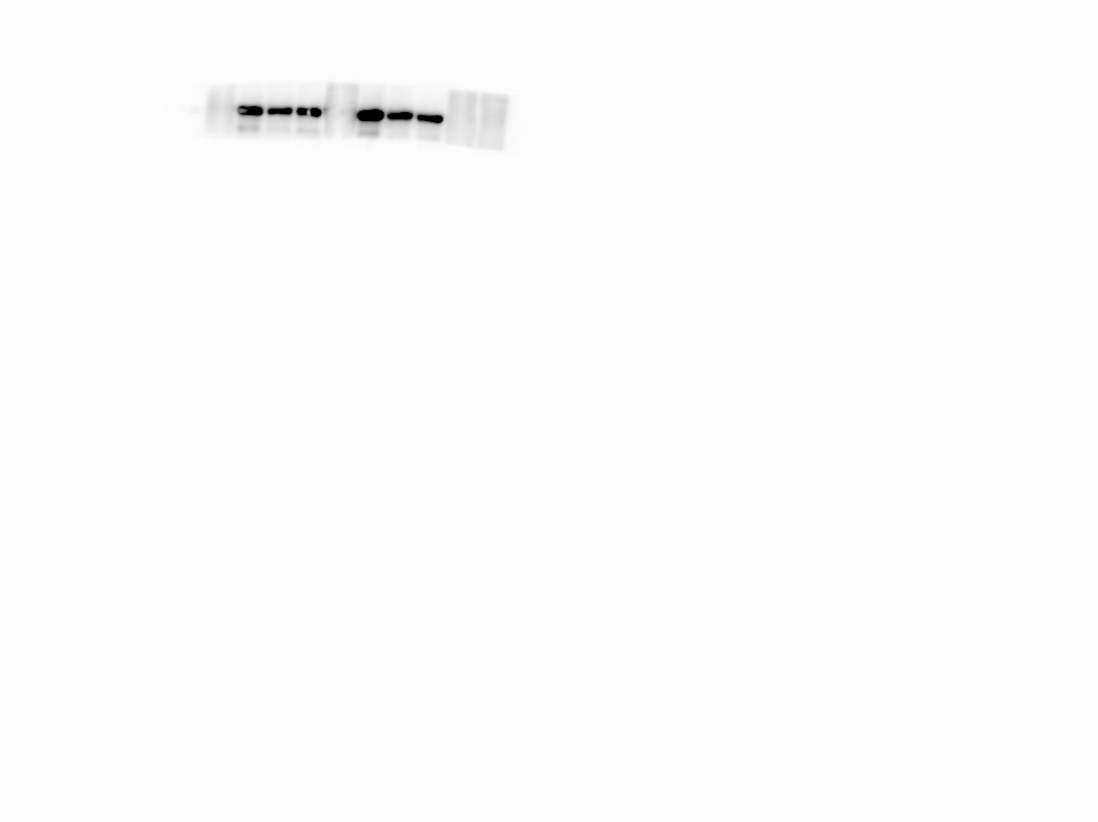

Bub1(mia)

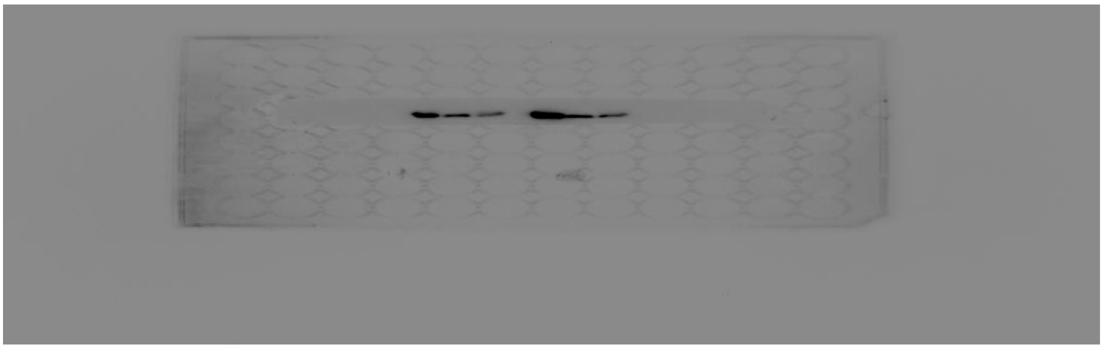

Actin(mia)

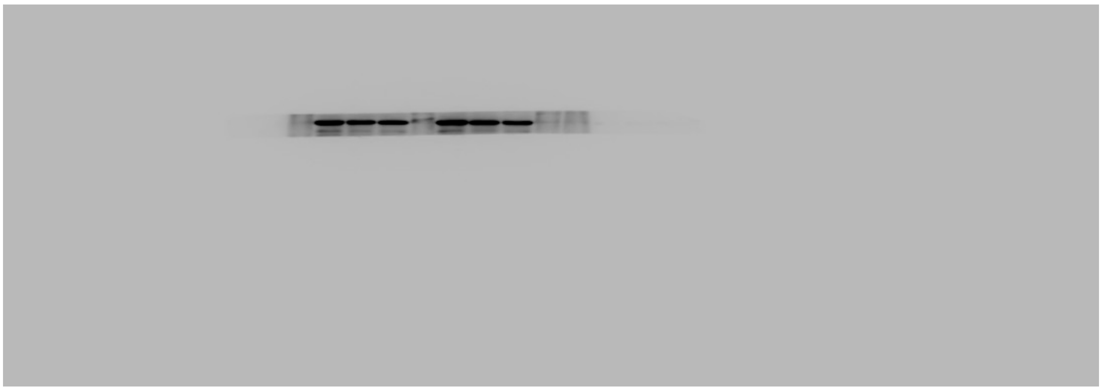

Bub1(pan1)

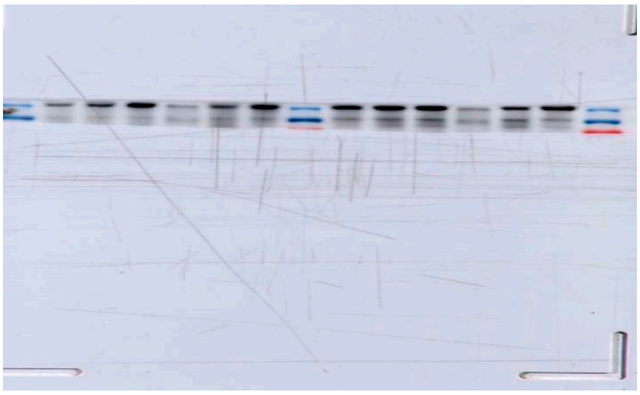

Actin(pan1)

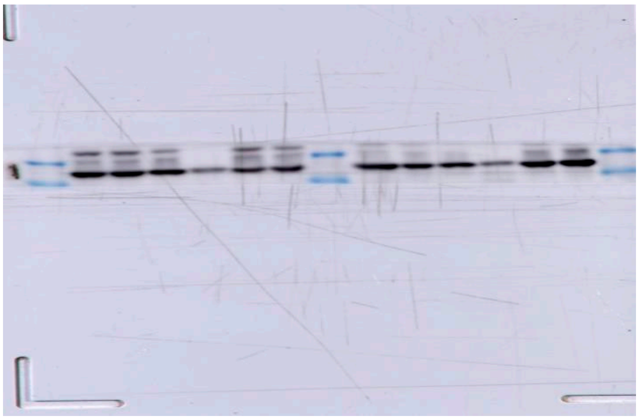

Figure 3a

Actin(pan Next 4 items)

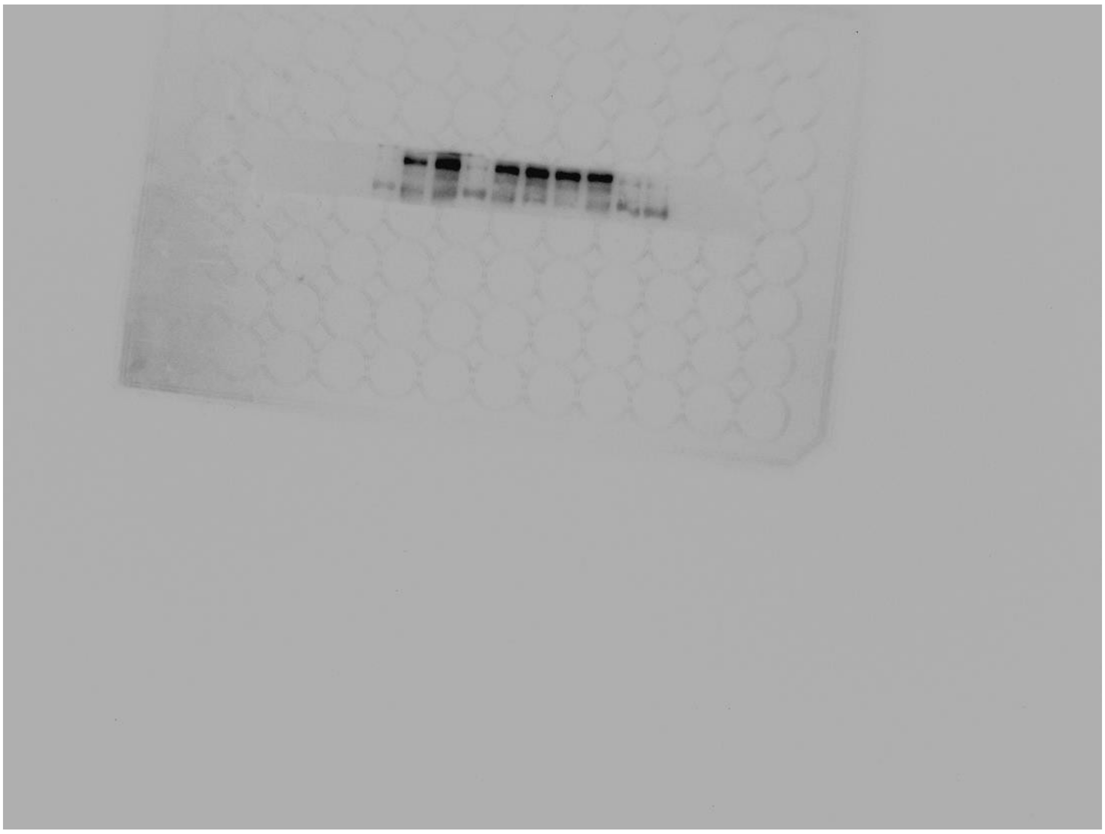

BUB1(pan1 Next 4 items)

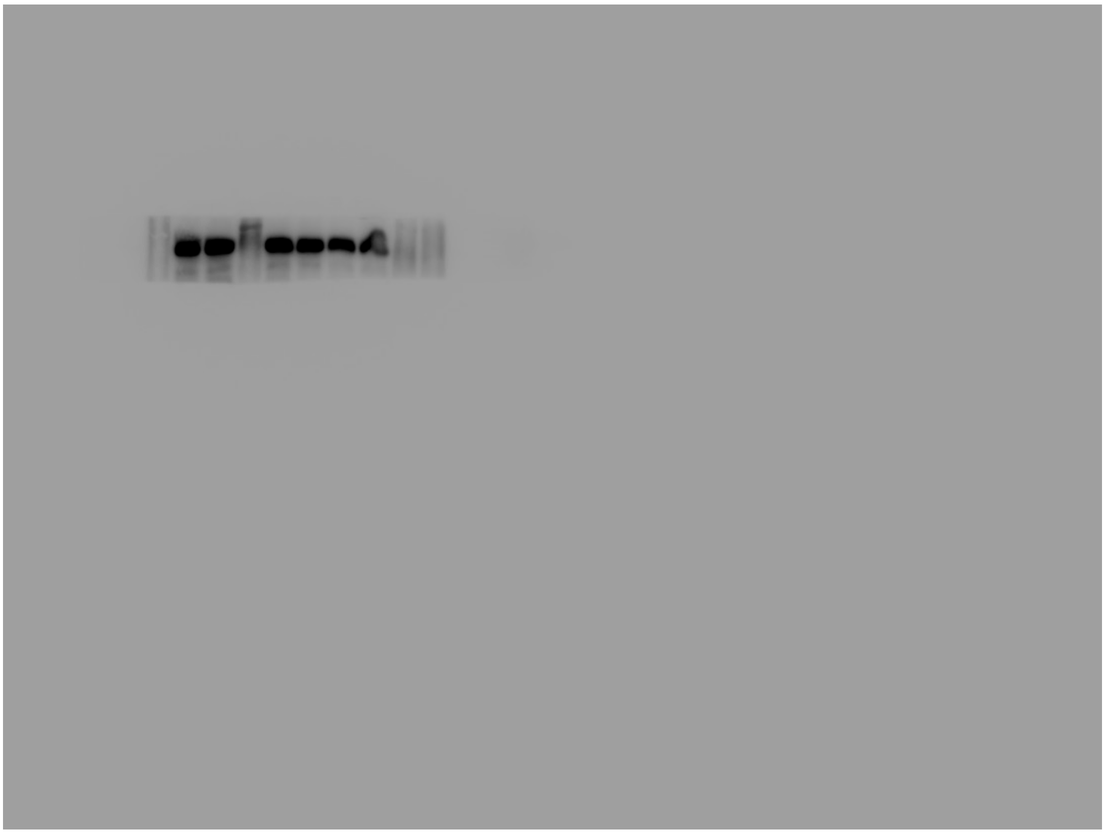

Actin(mia Next 4 items)

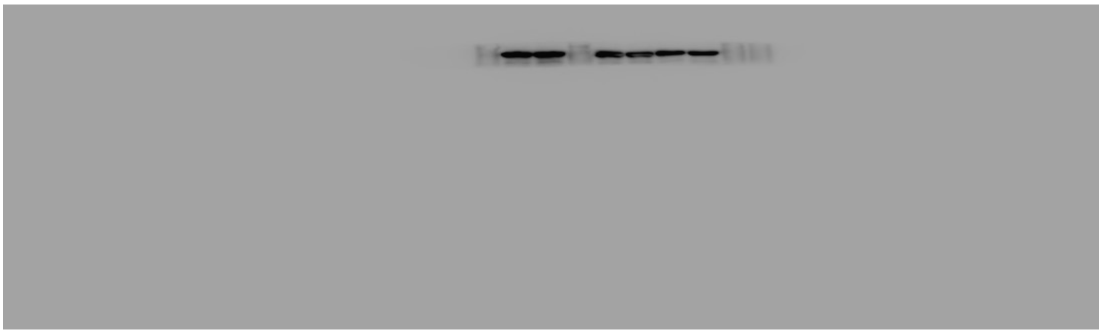

Bub1（mia Next 4 items）

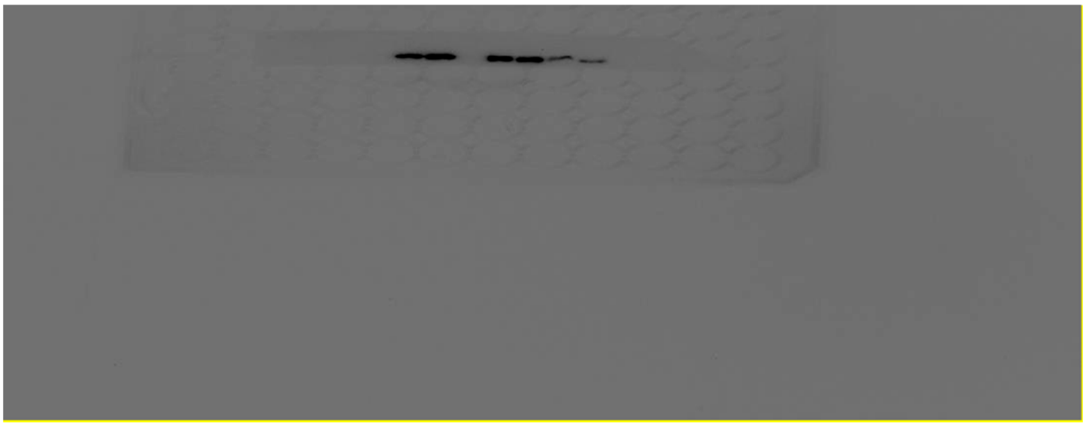

Due to the loss of the U disk after graduation of the first student who did this experiment, some of the above repeated experiment strips were missing.

Figure 4B

BUB1 (n=3)

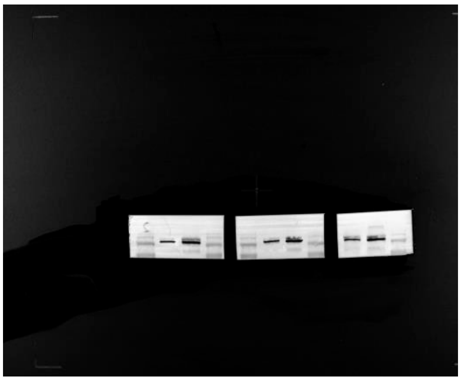

NF2 (n=3)

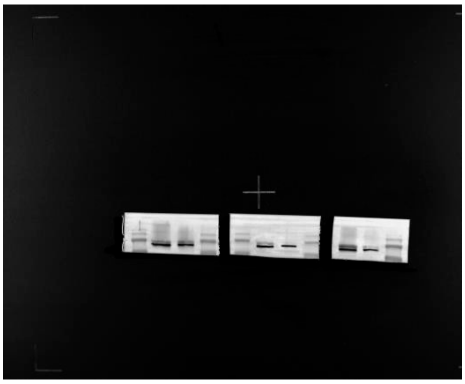

YAP (n=3)

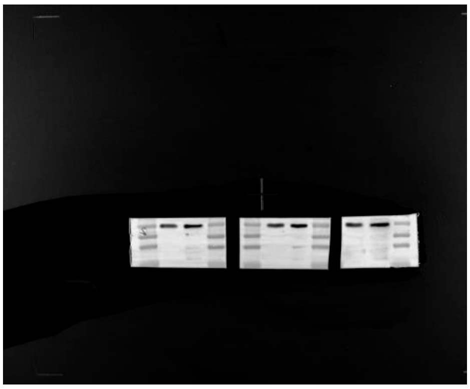

MOB1 (n=3)

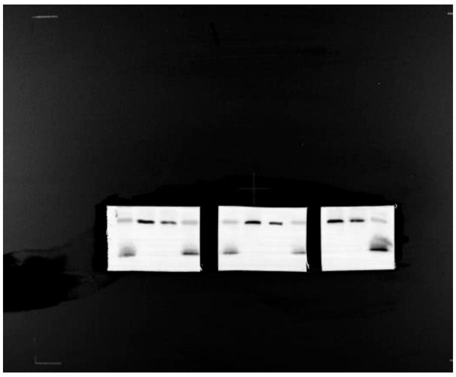

GAPDH (n=3)

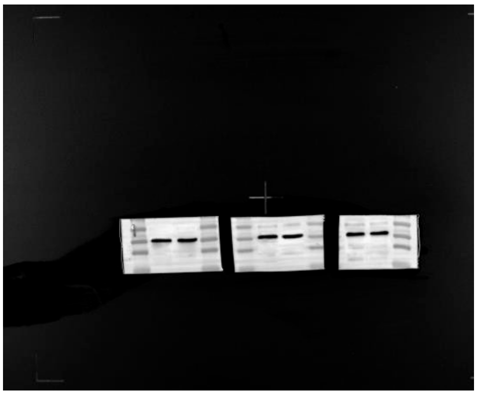

Figure 4E
BUB1 (n=3)

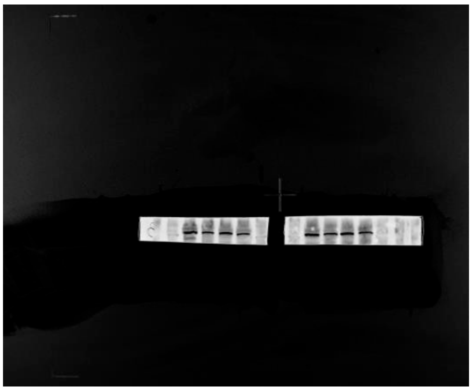

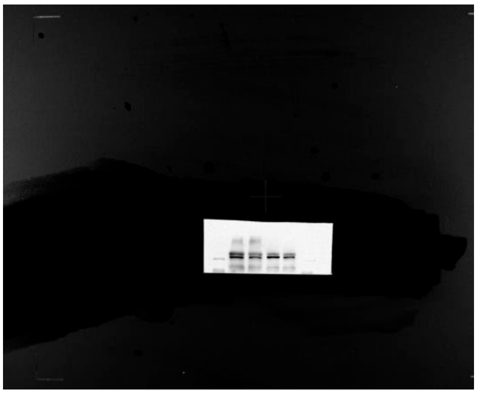

NF2 (n=3)

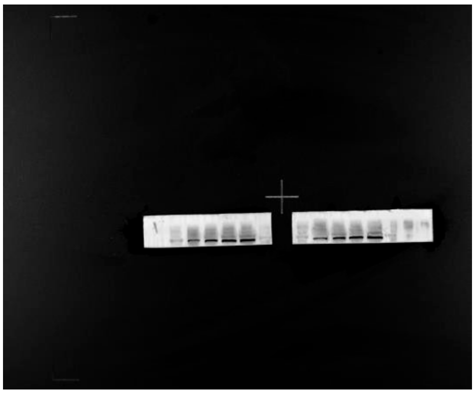

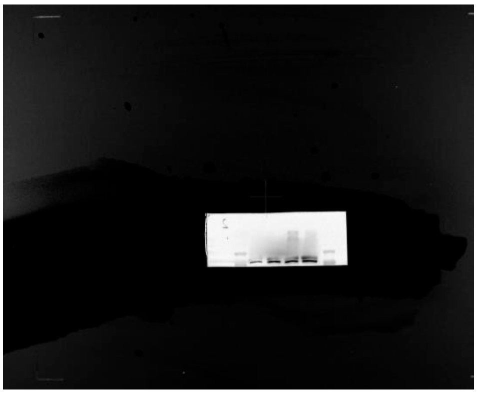

YAP (n=3)

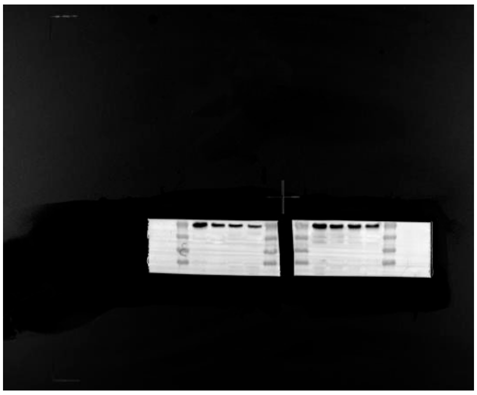

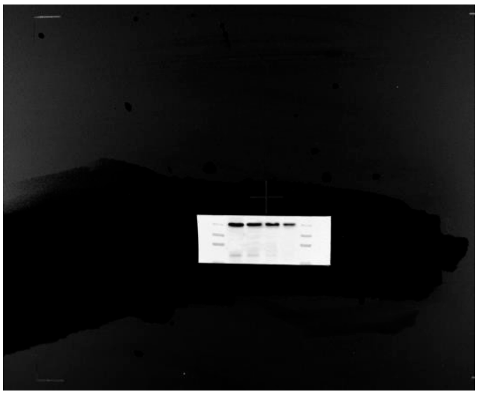

MOB1 (n=3)

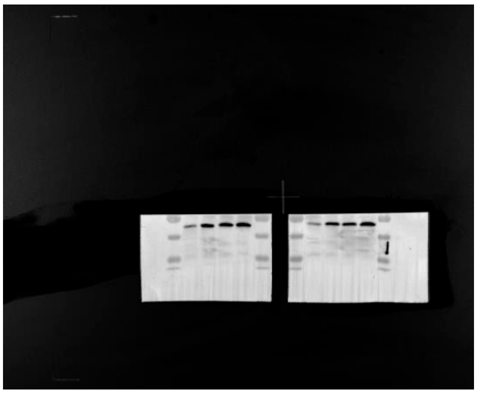

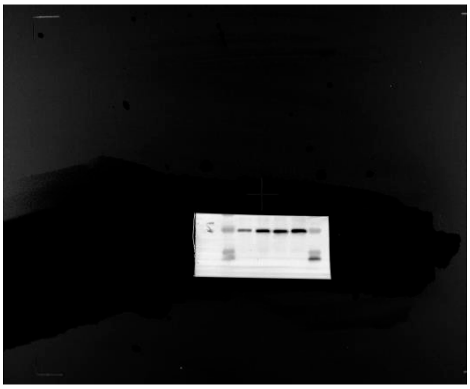

GAPDH (n=3)

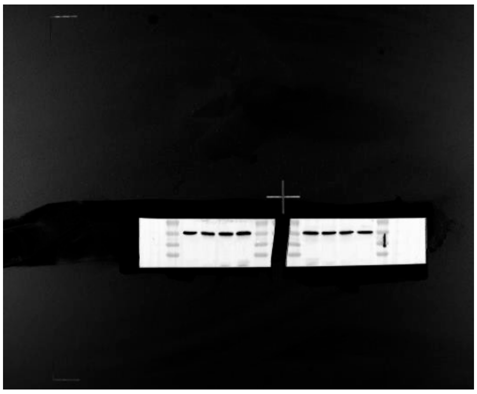

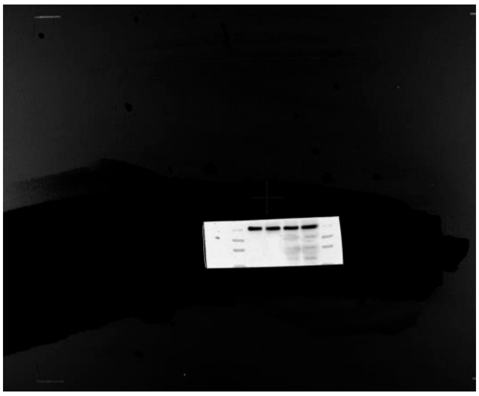

Figure5C
NF2 (n=3)

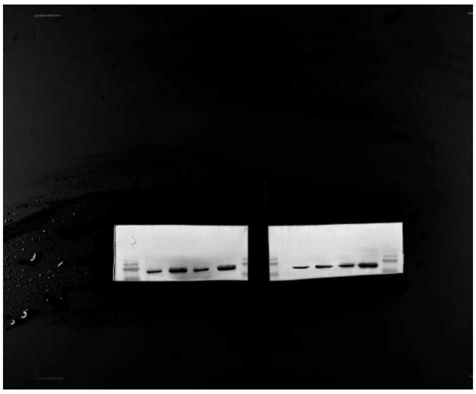

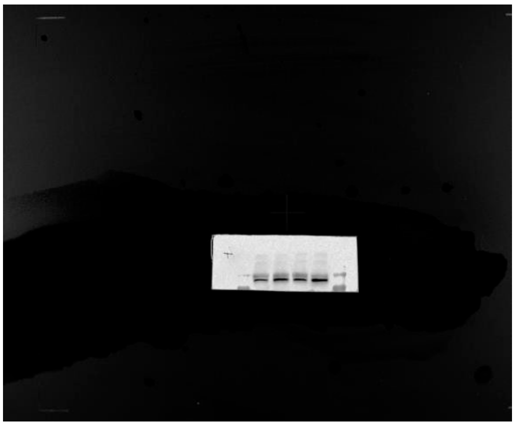

YAP (n=3)

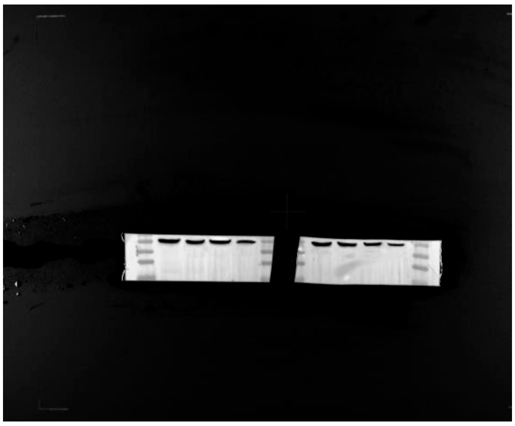

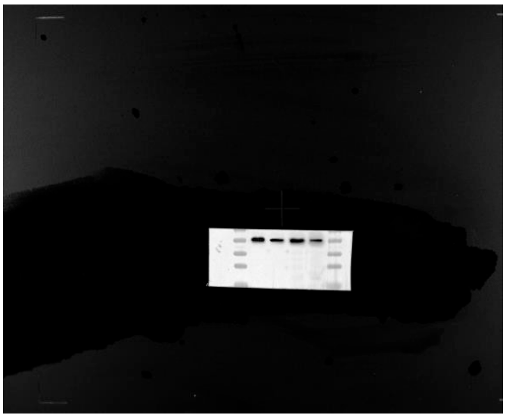

MOB1 (n=3)

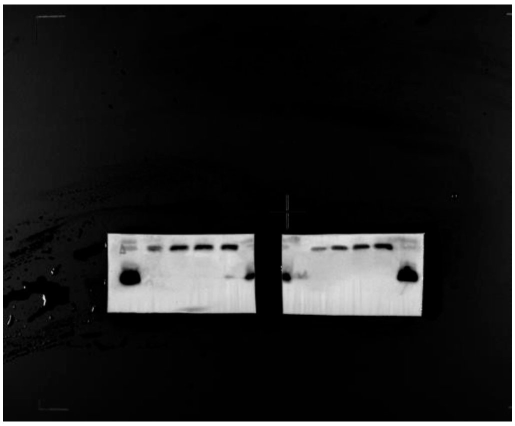

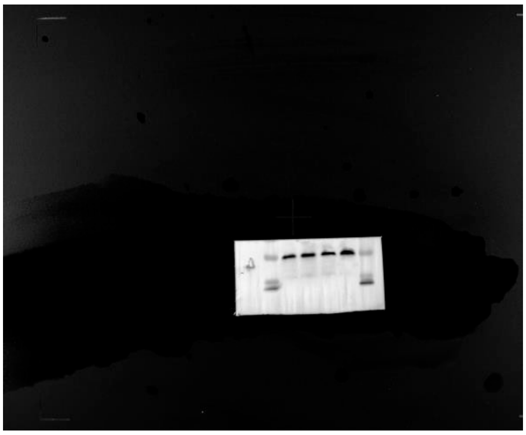

GAPDH (n=3)

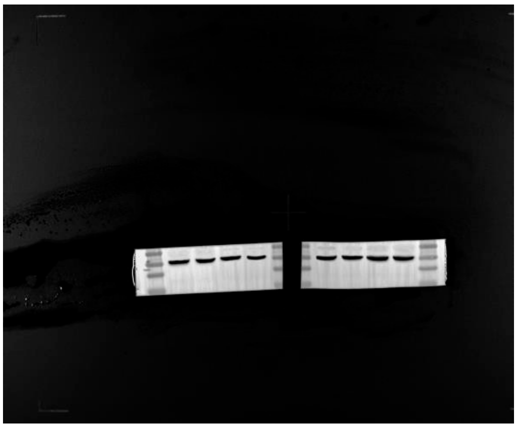

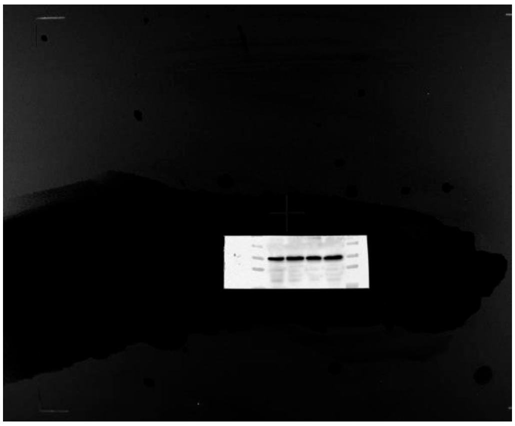

Supplement: Supplementary file 1 [file cancers-16-01540-s001.zip › cancers-2926397-supplementary.pdf]
